# Supplementary material for: Neural fatigue by passive induction: repeated stimulus exposure results in cognitive fatigue and altered representations in task-relevant networks
Source: Commun Biol. 2023 Feb 3;6:142. doi: 10.1038/s42003-023-04527-5 (PMC9898557; doi:10.1038/s42003-023-04527-5)
Supplement: Supplementary file 2 — Description of Additional Supplementary Files [file 42003_2023_4527_MOESM2_ESM.pdf]

Description of Additional Supplementary Files

File name: Supp Data 1
Description: This file contains the source data for Figure 1, being the mean accuracy (‘mean_Accuracy) in the texture discrimination task of each participant (‘SubNumber’), across experimental conditions (‘Session’ and ‘Quadrant’).

File name: Supp Data 2
Description: This file contains the source data for Figure 2b, being the accuracy (‘Accuracy’) of the MVPA classifier trained on the localizer data of each participant (‘SubNumber’), across experimental conditions (‘Session’ and ‘Quadrant’).

File name: Supp Data 3
Description: This file contains the source data for Figure 2c, being the accuracy (‘Accuracy’) of the MVPA classifier trained on the TDT data of each participant (‘SubNumber’), across experimental conditions (‘Session’ and ‘Quadrant’).

File name: Supp Data 4
Description: This file contains the source data for Figure 3b, being the baseline betas (‘BaselineBeta’) and change between sessions in classifying accuracy (‘DeltaAccuracy’) for each voxel within the subject-specific ROIs clustered by participant (‘SubNumber’) across experimental condition (‘Quadrant’).

File name: Supp Data 5
Description: This file contains the source data for Figure 3c, being the change, between sessions, in average activation within the subject-specific ROI (‘DeltaBeta’) and the random coefficients extracted from the model run on the behavioral data (‘RandomCoefficientsTDT’). Each row in the csv represents the data of a single participant.

File name: Supp Data 6
Description: This file contains the source data for Figure 4, being the change, between sessions, in self-reported sleepiness (‘DeltaKarolinska’) and the random coefficients extracted from the model run on the behavioral data (‘RandomCoefficientsTDT’). Each row in the csv represents the data of a single participant.
