# Supplementary material for: Neural fatigue by passive induction: repeated stimulus exposure results in cognitive fatigue and altered representations in task-relevant networks
Source: Commun Biol. 2023 Feb 3;6:142. doi: 10.1038/s42003-023-04527-5 (PMC9898557; doi:10.1038/s42003-023-04527-5)
Supplement: Supplementary file 9 — Reporting Summary [file 42003_2023_4527_MOESM9_ESM.pdf]

nature portfolio

Corresponding author(s): Stefano Ioannucci

Last updated by author(s): Oct 1, 2023

Reporting Summary

Nature Portfolio wishes to improve the reproducibility of the work that we publish. This form provides structure for consistency and transparency in reporting. For further information on Nature Portfolio policies, see our Editorial Policies and the Editorial Policy Checklist.

Statistics

For all statistical analyses, confirm that the following items are present in the figure legend, table legend, main text, or Methods section.

n/a	Confirmed	
☐	☒	The exact sample size (*n*) for each experimental group/condition, given as a discrete number and unit of measurement
☐	☒	A statement on whether measurements were taken from distinct samples or whether the same sample was measured repeatedly
☐	☒	The statistical test(s) used AND whether they are one- or two-sided<br>*Only common tests should be described solely by name; describe more complex techniques in the Methods section.*
☐	☒	A description of all covariates tested
☐	☒	A description of any assumptions or corrections, such as tests of normality and adjustment for multiple comparisons
☐	☒	A full description of the statistical parameters including central tendency (e.g. means) or other basic estimates (e.g. regression coefficient) AND variation (e.g. standard deviation) or associated estimates of uncertainty (e.g. confidence intervals)
☐	☒	For null hypothesis testing, the test statistic (e.g. *F*, *t*, *r*) with confidence intervals, effect sizes, degrees of freedom and *P* value noted<br>*Give P values as exact values whenever suitable.*
☒	☐	For Bayesian analysis, information on the choice of priors and Markov chain Monte Carlo settings
☒	☐	For hierarchical and complex designs, identification of the appropriate level for tests and full reporting of outcomes
☐	☒	Estimates of effect sizes (e.g. Cohen's *d*, Pearson's *r*), indicating how they were calculated

Our web collection on statistics for biologists contains articles on many of the points above.

Software and code

Policy information about availability of computer code

Data collection	Matlab 2019a
Data analysis	jamovi, SPM 12, Matlab 2019a

For manuscripts utilizing custom algorithms or software that are central to the research but not yet described in published literature, software must be made available to editors and reviewers. We strongly encourage code deposition in a community repository (e.g. GitHub). See the Nature Portfolio guidelines for submitting code & software for further information.

Data

Policy information about availability of data

All manuscripts must include a data availability statement. This statement should provide the following information, where applicable:

- Accession codes, unique identifiers, or web links for publicly available datasets
- A description of any restrictions on data availability
- For clinical datasets or third party data, please ensure that the statement adheres to our policy

All the data, scripts and analyses employed in this work can be found at the following links: https://zenodo.org/record/7395703, https://github.com/ste-ioan/nfbpi

Human research participants

Policy information about studies involving human research participants and Sex and Gender in Research.

Reporting on sex and gender	25 participants were recruited informally and took part voluntarily in the experiment (Mage = 21.6 ± 1.8, 10 m). They received 50€ in compensation for their participation.
Population characteristics	See above
Recruitment	See above
Ethics oversight	Local Ethical Review Board - Comité de protection des personnes Sud-Est V

Note that full information on the approval of the study protocol must also be provided in the manuscript.

Field-specific reporting

Please select the one below that is the best fit for your research. If you are not sure, read the appropriate sections before making your selection.

☐ Life sciences ☒ Behavioural & social sciences ☐ Ecological, evolutionary & environmental sciences

For a reference copy of the document with all sections, see nature.com/documents/nr-reporting-summary-flat.pdf

Behavioural & social sciences study design

All studies must disclose on these points even when the disclosure is negative.

Study description	quantitative
Research sample	Students from local facebook group 25 participants were recruited informally and took part voluntarily in the experiment (Mage = 21.6 ± 1.8, 10 m)
Sampling strategy	random, effect-size estimation from previous study
Data collection	eye tracker, pen and paper, fMRI. Scanner technician was present, no blinding procedure.
Timing	July 2021 to September 2021
Data exclusions	2 exclusions: one fMRI data-set due to distortions and one behavioral data-set due to failure to record responses.
Non-participation	Surprisingly none.
Randomization	Random, based on participant number (evens were assigned to one condition, odds to another).

Reporting for specific materials, systems and methods

We require information from authors about some types of materials, experimental systems and methods used in many studies. Here, indicate whether each material, system or method listed is relevant to your study. If you are not sure if a list item applies to your research, read the appropriate section before selecting a response.

Materials & experimental systems

n/a	Involved in the study	
☒	☐	Antibodies
☒	☐	Eukaryotic cell lines
☒	☐	Palaeontology and archaeology
☒	☐	Animals and other organisms
☒	☐	Clinical data
☒	☐	Dual use research of concern

Methods

n/a	Involved in the study	
☒	☐	ChIP-seq
☒	☐	Flow cytometry
☐	☒	MRI-based neuroimaging

Magnetic resonance imaging

Experimental design

Design type
Event and block design.

Design specifications
Event : 4 blocks, with equal numbers of trials per quadrant and target alignment, lasting ~15 minutes on average (~2 seconds per trial).
Block : 6 times for 12 seconds, alternating. The total duration of a localizer session was ~5 minutes.

Behavioral performance measures
Button press, accuracy in response (binary choice).

Acquisition

Imaging type(s)
Functional.

Field strength
3T

Sequence & imaging parameters
All the functional MRI series were acquired using a 2D simultaneous multi-slice echo gradient echo planar sequence (2 x 2 mm voxels in-plane; 2 mm slice thickness with no gap; 40 transverse slices, 170 x 170 mm field-of-view; matrix 86 x 86; partial-fourier 6/8; repetition time = 1 s; echo time = 31 ms; multiband slice acceleration factor of 4; phase encoding direction Anterior-Posterior; flip angle 71°; bandwidth 1384 hz/pixel).

Area of acquisition
The acquisition was aligned to the calcarine scissure of the participants, whole-brain.

Diffusion MRI ☐ Used ☒ Not used

Preprocessing

Preprocessing software
FSL and SPM.

Normalization
Each series was realigned to the first localizer acquisition using rigid transformation with FSL flirt using mean as reference image and normalized to MNI space using SPM 12 by warping the T1 anatomical scans and applying the linear transformations to the functional scans.

Normalization template
MNI

Noise and artifact removal
Susceptibility-induced distortions were estimated and corrected for all functional images using the top-up method with FSL6.0.3. In a second step, they were motion-corrected using linear transformation with the automated tool MCFLIRT in FSL and intensity-scaled to 1000. Then, the PhysIO Toolbox with MATLAB v9.7 was used to apply physiological noise correction. Both cardiac and respiratory signal were recorded using a finger pulse oximeter and a pneumatic belt during MRI acquisition. Respective physiological regressors were created using the RETROICOR algorithm as well as heart rate variability and respiratory volume per time and regressed out of from the functional MRI time series.

Volume censoring
N/A.

Statistical modeling & inference

Model type and settings
Multivariate and Univariate.

Effect(s) tested
For each subject, a contrast was carried out on their localizer data in both sessions (baseline and conclusion) between the targets in one quadrant versus the other (e.g. Left Quadrant (LQ) > Right Quadrant (RQ) and viceversa). Then, the clusters surviving Family Wise Error multiple comparison correction (pfwe < 0.05) in both localizer sessions (AND operation) were combined into a single, subject-specific, binarized functional ROI. Therefore, for each subject we obtained two ROIs, one for each side of quadrant stimulation, which were later categorized as saturated or non saturated, depending on which quadrant would display the stimuli during stimulation. MVPA was carried out both on the localizer beta coefficients and the TDT beta coefficients, with the same LQ vs RQ contrasts mentioned above as regressor. In the case of the localizer, the beta coefficients were derived by means of separate regressors for each single 12-second block of stimulation, while in the case of the TDT, regressors included groups of 10 successive same-condition trials.
MVPA analysis was carried out via The Decoding Toolbox, in the baseline and conclusion sessions, separately for the RQ and LQ ROIs. The classifier was asked, with a leave-one-out cross-validation procedure, to label in which quadrant were the stimuli presented, given the observed brain activity. Therefore, for each participant, we derived a data point of classifier accuracy for each quadrant, at baseline and conclusion, for both the localizer and the TDT brain scans. These were employed in a repeated measures ANOVA.
Additionally, to estimate a voxel-wise measure of classifying accuracy, the above analysis was repeated with a searchlight procedure using a 4 voxel radius within the subject-specific ROIs, and the resulting voxel-wise accuracy measures were employed in a linear mixed model analysis.

Specify type of analysis: ☐ Whole brain ☐ ROI-based ☒ Both

Anatomical location(s)
Functional localizer.

Statistic type for inference<br>(See Eklund et al. 2016)	Cluster-wise with nonparametric permutation test for univariate analysis, Cluster-wise for functional ROI determination which were then used in MVPA to derive the measures of accuracy tested in the rANOVA.
Correction	FWE.

Models & analysis

n/a	Involved in the study
☒	☐ Functional and/or effective connectivity
☒	☐ Graph analysis
☐	☒ Multivariate modeling or predictive analysis

Multivariate modeling and predictive analysis	See above (effects tested).
